# Case report of aggressive treatments for large-cell neuroendocrine carcinoma of the esophagus

**DOI:** 10.1016/j.ijscr.2019.06.056

**Published:** 2019-06-28

**Authors:** Yosuke Nakao, Tetsuya Okino, Yo-ichi Yamashita, Katsunobu Taki, Shigeki Nakagawa, Katsutaka Matsumoto, Mataro Goto, Hideo Baba

**Affiliations:** a Department of Surgery, National Hospital Organization Miyakonojo Medical Center, Miyazaki, Japan; b Department of Gastroenterological Surgery, Graduate School of Life Sciences, Kumamoto University, Kumamoto, Japan

**Keywords:** Neuroendocrine carcinoma, Large cell, Esophagus, Case report

## Abstract

**Introduction:**

Neuroendocrine carcinoma of the esophagus is a rare and highly aggressive disease, and the biological features are poorly understood.

**Presentation of case:**

We report a case of large-cell neuroendocrine carcinoma of the esophagus in a 73-year-old male patient with aggressive surgical treatment. Upper gastrointestinal endoscopy revealed an esophageal large mass. Histological examination of biopsy specimens indicated a neuroendocrine carcinoma. First, we performed subtotal esophagectomy with lymphadenectomy, but he experienced metastasis at the liver and rib 5 months later. Next, we performed partial hepatectomy and radiotherapy for rib metastasis, but he experienced metastasis at the left pulmonary hilar lymph node and sacral bone. Finally, we performed chemotherapy using cisplatin and irinotecan.

**Discussion:**

The therapeutic strategy for large-cell NEC of the esophagus is unestablished. Thus, accumulating the therapeutic results garnered from various treatment tools is considerably important.

**Conclusion:**

Aggressive multimodal treatments including surgery have a possibility to gain better survival in patients with large-cell NEC of the esophagus.

## Highlights

• Neuroendocrine carcinoma of the esophagus is a rare and highly aggressive disease.
• A case of large-cell neuroendocrine carcinoma of the esophagus in a 73-year-old male patient with aggressive surgical treatment.
• We performed subtotal esophagectomy, partial hepatectomy, radiotherapy and chemotherapy using cisplatin and irinotecan.
• We believe that aggressive treatment can become one treatment option with the aim of extending survival.

## 1 Introduction

Neuroendocrine carcinoma (NEC) is a rare disease with a reported incidence of between 0.4% and 2% among all malignancies of the esophagus [[1], [2], [3]]. NEC is categorized into two morphological types: small-cell and large-cell. The former is the dominant histological type and only 10% of NEC is classified as large-cell [4].

At this time, only a small number of retrospective studies with small cohorts and several case reports covering treatment outcomes of large-cell NEC of the esophagus are available [1,[5], [6], [7], [8]]. The reported prognostic outcome is poor because of its high malignant potential [5]. Several comparatively long-term survivors were reported who had been treated by surgery or surgery plus adjuvant chemotherapy [6,7]. In addition, radiotherapy was also reported to be expected to extend the survival outcome [9,10]. However, the optimal treatment strategy for large-cell NEC of the esophagus remains unestablished owing to its rarity [10,11]. Thus, accumulating the therapeutic results garnered from various treatment tools is considerably important.

Herein, we report a case of large-cell NEC of the esophagus in a patient who underwent aggressive multimodal treatments and who attained the longest survival among patients previously reported.

This work has been reported in line with the SCARE criteria [12].

## 2 Presentation of case

**Fig. 1:**
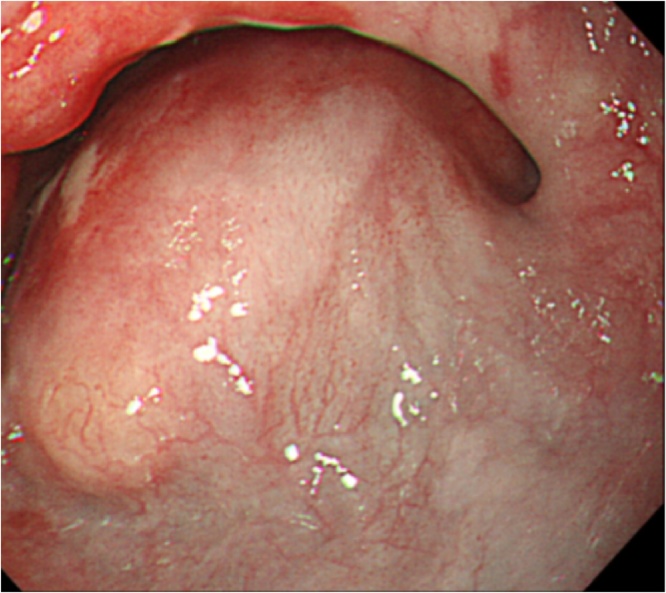
Upper gastrointestinal endoscopy revealed an esophageal large mass.

**Fig. 2:**
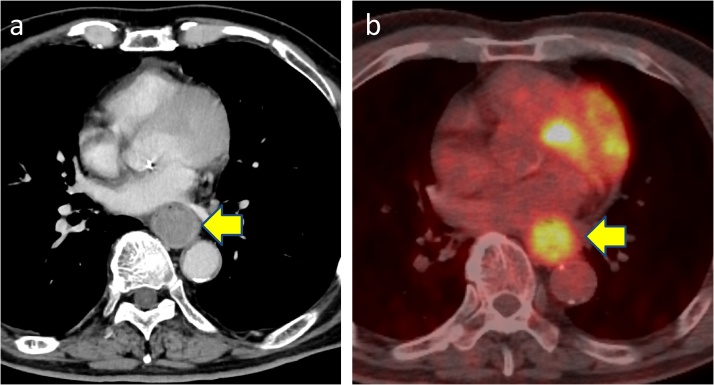
**a**) CT scan showed large mass in the lower esophagus. **b**) PET-CT showed a hypermetabolic esophageal tumor (SUV_max_ = 7.89).

A 73-year-old male patient arrived at our hospital reporting difficulty in swallowing. He had no pain in swallowing, no heartburn and no nausea. He lost approximately 4 kg from a baseline weight of 75 kg despite having a normal appetite. He had history of smoking (Brinkman Index = 1200). There was no family history of gastrointestinal malignancies. Upper gastrointestinal endoscopy revealed a large esophageal mass with normal mucosa on the surface at 33 cm distant from an incisor tooth (Fig. 1). NEC was pathologically confirmed from the biopsy specimens. Computed tomography (CT) showed a poorly enhanced large mass in the lower esophagus (Fig. 2a). Positron emission tomography/computed tomography (PET-CT) revealed a hypermetabolic esophageal tumor (standardized uptake value max = 7.89) with no evidence of distant metastasis (Fig. 2b).

**Fig. 3:**
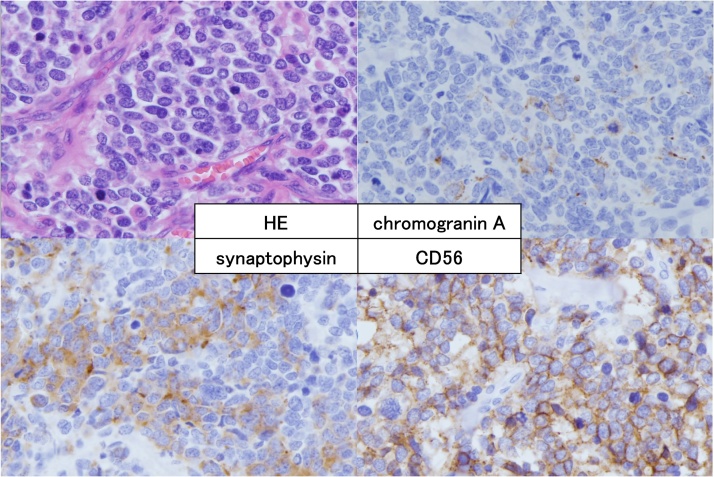
Immunohistochemical findings. Tumor cells were reactive with synaptophysin, chromogranin A stain, and CD56.

**Fig. 4:**
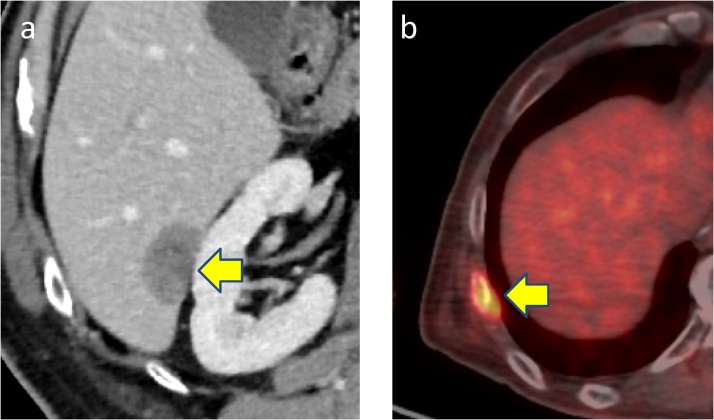
**a**) CT scan showed metastasis in S6 of the liver. **b**) PET-CT showed metastasis in the right eighth rib.

**Fig. 5:**
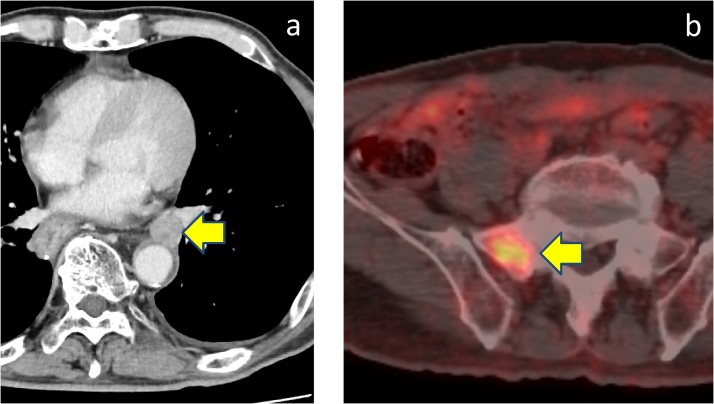
PET-CT showed new metastasis in hilar lymph node and sacrum.

We performed open subtotal esophagectomy with lymphadenectomy. The patient was discharged on postoperative day 31 with no serious complications. Histopathological examination showed a NEC of the large-cell type in view of irregular strands of large polygonal cells. In addition, synaptophysin, chromogranin A and CD56 were positive (Fig. 3) and the Ki-67 labeling index was 80% by immunohistochemical staining. The pathological stage was pT1b pN2 M1, pStage IV. We performed adjuvant chemoradiation therapy (total 45 Gy/25 fr) with combined chemotherapy that consisted of docetaxel (60 mg/m^2^ on day 1), fluorouracil (350 mg/m^2^ on days 1–5) and cisplatin (6 mg/m^2^ on days 1–5) for two cycles after the operation. However, 5 months after the operation, a CT scan showed metastasis in segment 6 of the liver (Fig. 4a). Furthermore, PET-CT showed metastasis in the right eighth rib (Fig. 4b). We then performed open partial hepatectomy for the liver metastasis. The patient was discharged on postoperative day 10 with no serious complications. Then, we performed radiation therapy (total 51 Gy/17 fr) for the rib metastasis. The metastasis at the right eighth rib disappeared; however, 2 months after radiation therapy, PET-CT showed a new metastasis of a hilar lymph node in the left lung (Fig. 5a) and sacral bone (Fig. 5b). We started chemotherapy with cisplatin (60 mg/m^2^ on day 1) and irinotecan (60 mg/m^2^ on days 1, 8, and 15). After two cycles of chemotherapy, the size of the left lung hilar lymph node reduced from 24 mm to 17 mm. Finally, after 7 months from the start of the last chemotherapy (20 months after subtotal esophagectomy), the patient died of disease progression.

## 3 Discussion

Large-cell NEC of the esophagus is extremely rare. With regard to its clinical course, scant previous studies are available [1,13]. Specific symptoms of large-cell NEC do not exist and patients usually present with various symptoms, such as chest pain, dysphagia, odynophagia, and weight loss as with other malignancies in the esophagus. A typical case shows a single lesion, which commonly develops in the lower third in the esophagus. This is because neuroendocrine cells are mainly distributed in mucosal glands of the distal esophagus [1,14,15]. Our case showed similar clinical findings to those previously reported.

The therapeutic strategy for large-cell NEC of the esophagus is unestablished. Only one retrospective study suggested that multimodality treatments including surgery, chemotherapy, and radiotherapy may be useful, but only when the tumor is a resectable limited disease [10]. However, the optimal treatment for unresectable and recurrent cases after surgery remains unclear.

In the present case, we performed aggressive treatments including subtotal esophagectomy, adjuvant chemoradiotherapy, hepatectomy for liver metastasis, radiation therapy for rib metastasis and chemotherapy for hilar lymph node metastasis in the left lung and sacral bone metastasis. Each treatment was locally effective. Although each new metastatic site rapidly appeared, the patient survived 20 months because of this aggressive approach. This is the longest any patient has survived compared with previous reported cases with extensive large-cell NEC of the esophagus [3,8,16,17].

Although the patient in the current case survived for a longer time than any other previously reported, we considered that more effective treatments are necessary to improve the therapeutic outcome of this refractory malignancy. Notably, a novel chemotherapeutic agent is desirable to prolong survival in patients with advanced NEC. At this time, chemotherapy with cisplatin and irinotecan is first considered for extensive or metastatic NEC [18,19]. Chemotherapy with cisplatin and etoposide can also become treatment options [19,20]. Recently, Kasahara et al. documented a retrospective study with 18 patients who underwent amrubicin monotherapy after failure of platinum-based chemotherapy [21]. In this study, the overall response rate and progression free survival were reported as 11.1% and 4.0 month, respectively. To further improve treatment outcomes for this refractory malignancy, several influential studies have been conducted. Christopoulos et al. reported the effectiveness of everolimus with paclitaxel and carboplatin as the first-line treatment for metastatic large-cell neuroendocrine lung carcinoma via a multicenter phase II trial [22]. In addition, the PRODIGE 41-BEVANEC study using bevacizumab in combination with FOLFILI after the failure of a platinum-etoposide regimen in patients with advanced NEC is ongoing [23].

## 4 Conclusion

Consequently, we believe that aggressive treatment can become one treatment option with the aim of extending survival in patients with advanced large-cell NEC of the esophagus. In addition, we consider that further accumulation of treatment experiences for large-cell NEC of the esophagus is very important to improve treatment outcomes of this refractory malignancy.

Authors are Yosuke Nakao, Tetsuya Okino, Yo-ichi Yamashita, Katsunobu Taki, Shigeki Nakagawa, Katsutaka Matsumoto, Mataro Goto and Hideo Baba.

## Conflicts of interest

The authors declare that they have no conflicts of interest.

Authors are Yosuke Nakao, Tetsuya Okino, Yo-ichi Yamashita, Katsunobu Taki, Shigeki Nakagawa, Katsutaka Matsumoto, Mataro Goto and Hideo Baba.

## Funding

This study did not receive any funding support.

## Ethical approval

It was deemed to be unnecessary for this report.

## Consent

This paper is a case report. Written informed consent was obtained from the patient.

## Author contribution

Hiroshi Sawayama: writing the manuscript, managed a patient.

Tetsuya Okino: managed a patient, review, and/or revision of the manuscript.

Yo-ichi Yamashita: managed a patient, review, and/or revision of the manuscript.

Katsunobu Taki: managed a patient, review, and/or revision of the manuscript.

Shigeki Nakagawa: managed a patient, review, and/or revision of the manuscript.

Katsutaka Matsumoto: managed a patient, review, and/or revision of the manuscript.

Mataro Goto: review, and/or revision of the manuscript.

Hideo Baba: study supervision.

## Registration of research studies

Hideo Baba.

## Guarantor

The Guarantor of this paper is Hideo Baba.

## Provenance and peer review

Not commissioned, externally peer-reviewed.
